# Dynamic tissue-specific mosaicism in constitutional non-supernumerary ring chromosome 21 with a Down syndrome-like phenotype: a case report and genotype-phenotype analysis

**DOI:** 10.3389/fgene.2026.1958717

**Published:** 2026-10-26

**Authors:** Daniel Martínez Anaya, Alejandro Zinser Sainas, Marelen Cruz Cruz, Ana Lucía Yáñez-Félix, Consuelo Salas Labadía, Carlos Andre Orta Barcenas, David Arturo Sosa Sánchez, Emiy Yokoyama Rebollar, Patricia Pérez-Vera

**Affiliations:** 1 Genetics and Cancer Laboratory, National Pediatric Institute, Mexico City, Mexico; 2 Department of Human Genetics, National Pediatric Institute, Mexico City, Mexico; 3 Specialized Genetic Analysis Laboratory Mexico (LAGEM), Mexico City, Mexico

**Keywords:** complex genomic imbalances, constitutional non-supernumerary ring chromosome 21, dosage-sensitive genes, down syndrome-like phenotype, genotype–phenotype correlations, partial monosomy 21q, partial trisomy 21q, tissue-specific dynamic mosaicism

## Abstract

**Introduction:**

Ring chromosome 21 (r (21)) is a rare constitutional rearrangement involving terminal deletions and significant phenotypic heterogeneity. High-resolution genomic analyses have demonstrated that ring chromosomes may harbor complex genomic imbalances and dynamic mosaicism. However, the contributions of ring chromosome instability, tissue-specific mosaicism, and complex dosage imbalance to the clinical phenotype remain poorly understood.

**Case Presentation:**

We report an 8-month-old boy with developmental delay, congenital heart disease, hearing impairment, structural brain abnormalities, and dysmorphic features suggestive of Down syndrome (DS), although lacking the complete classical phenotype. Cytogenomic analyses identified a constitutional non-supernumerary r (21) associated with a 18.77-Mb duplication of 21q21.2-q22.3 and a 3.09-Mb terminal deletion of 21q22.3. Fluorescence *in situ* hybridization (FISH) resolved the ring chromosome structure, identifying four instability-derived clones, and demonstrated tissue-specific mosaicism across lymphocytes, oral mucosa epithelial cells, and urinary sediment epithelial cells, with a duplicated monocentric r (21) as the predominant clone. Comparison with the four previously reported constitutional non-supernumerary r (21) cases revealed a recurrent combination of Down syndrome critical region (DSCR) duplication and terminal 21q deletion, despite substantial phenotypic variability.

**Discussion and Conclusion:**

This study expands the clinical and cytogenomic spectrum associated with constitutional non-supernumerary r (21). Tissue-specific mosaicism and recurrent genomic imbalances suggest that the DS-like phenotype cannot be explained solely by dosage alterations involving the classical or highly restricted DSCR. Our findings suggest a mechanism in which dynamic ring chromosome instability, and the cumulative dosage imbalance of multiple chromos​ome 21 genes, contribute to the phenotypic variability observed in these patients. Additional well-characterized cases are essential for refining the genotype–phenotype correlations of this rare genomic disorder.

## 1 Introduction

Ring chromosome 21 (r (21)) syndrome (ORPHA: 1445) is a rare genomic disorder. Among the reported patients with ring chromosome syndrome between 1962 and 2022, r (21) accounts for approximately 7.1% of cases worldwide (Li et al., 2022). Clinical manifestations are highly heterogeneous and may include developmental delay, intellectual disability, epilepsy, growth impairment, congenital malformations, acute B-cell lymphoblastic leukemia, infertility, and dysmorphic features (Chen et al., 2012; Guzmán-Santiago et al., 2025).

The clinical variability observed among patients with constitutional r (21) reflects the combined effects of several pathogenic mechanisms. These include mitotic instability of the ring chromosome, gene dosage imbalance resulting from segmental deletions and duplications, and tissue-specific mosaicism. Consequently, individuals carrying apparently similar ring chromosomes may exhibit markedly different clinical manifestations. Depending on the genomic content of the rearranged chromosome, constitutional r (21) may result in either a classic Down syndrome (DS) phenotype or a more complex DS-like phenotype caused by concurrent chromosome 21 copy number imbalances (Chen et al., 2012; Zhang et al., 2012; Ambulkar et al., 2023).

Despite advances in molecular cytogenetic technologies, the genotype–phenotype correlations underlying constitutional non-supernumerary r (21) remain poorly understood. Cases combining terminal 21q deletions, large intrachromosomal duplications encompassing the Down syndrome critical region (DSCR), and widespread dynamic mosaicism are exceptionally rare. Consequently, the contribution of ring chromosome instability and dosage-sensitive genes located both within and outside the DSCR to the resulting phenotype remains largely unexplored (Chen et al., 2012; Zhang et al., 2012; Ambulkar et al., 2023).

Here, we describe the clinical, cytogenetic, and cytogenomic characterization of a patient with a constitutional non-supernumerary r (21) associated with a DS-like phenotype. The complex ring chromosome structure was resolved by combining G-banding, SNP-array and fluorescence *in situ* hybridization (FISH) analyses using multiple chromosome 21-specific probes. The tissue-specific distribution of ring chromosome-derived clones was subsequently investigated by interphase FISH in tissues representing different embryonic germ layers. Finally, we performed a systematic literature review of comparable cases together with genomic database analyses to investigate genotype–phenotype correlations and further delineate the clinical spectrum associated with this rare chromosomal disorder.

## 2 Clinical description

The proband is an 8-month-old male with dysmorphic features, intellectual disability, and developmental delay who was the first full-term child of healthy non-consanguineous parents. His mother was 27 years old, and his father was 42 years old at the time of his birth. Family history and prenatal course were unremarkable. He was delivered by cesarean section at 40 weeks of gestation. His birth weight was 2.36 kg (Z = -1.66), length was 48 cm (Z = -0.92) and Apgar scores were 8/9.

He was referred to the National Institute of Pediatrics (Mexico City, Mexico) during the first 2 months of life because of intestinal atresia. Exploratory laparotomy revealed a Meckel diverticulum, which was surgically resected. During this period, dysmorphic features and a systolic murmur were noted, prompting referral to the Department of Human Genetics.

At evaluation, his weight was 2.87 kg (Z = −2.8), length was 48 cm (Z = -3.36), and head circumference was 33.5 cm (Z = −4.6). Physical examination revealed microcephaly, a broad forehead with bitemporal narrowing, sparse eyebrows, upslanting palpebral fissures, bilateral epicanthal folds, telecanthus, malar flattening, anteverted nares, a thin upper lip vermilion, downturned corners of the mouth, low-set ears, and a low posterior hairline. Examination of the extremities showed persistent fetal fingertip pads, a slightly marked single transverse palmar crease and abnormal plantar creases (Figures 1A–D).

**FIGURE 1:**
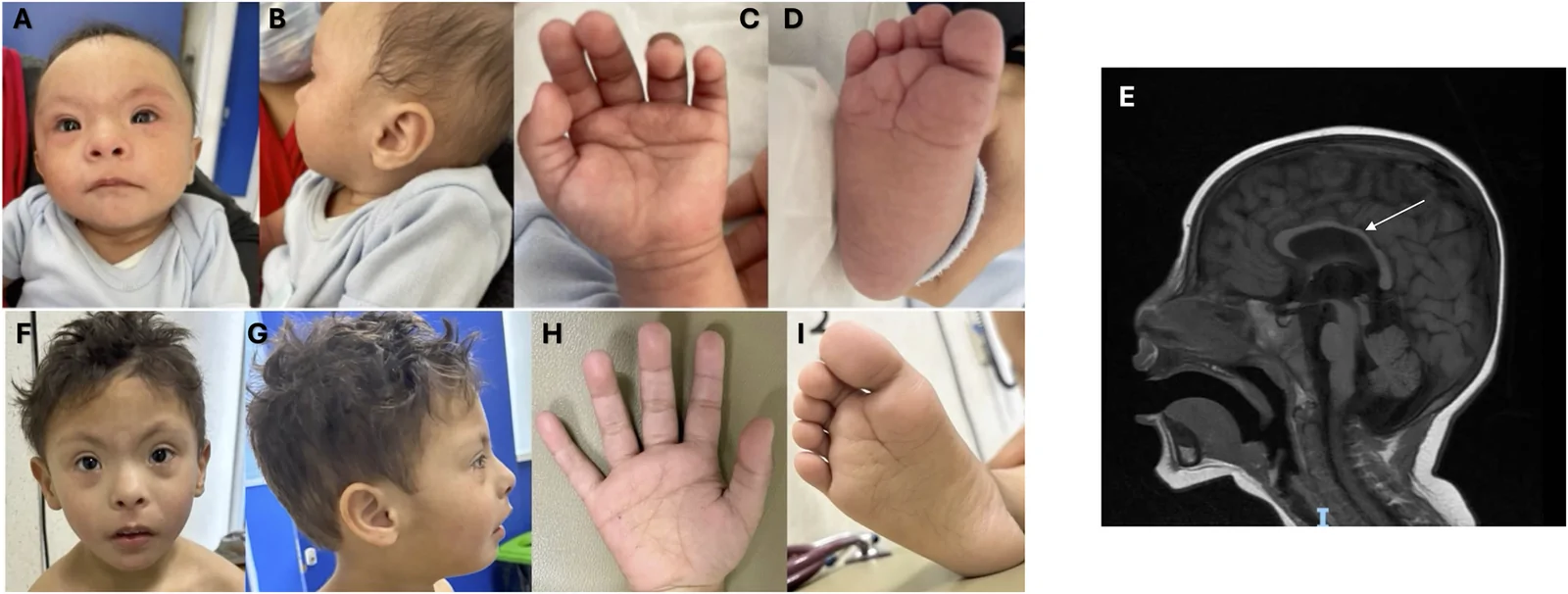
Clinical features and neuroimaging findings of the proband. **(A–D)** Clinical photographs obtained at 8 months of age. **(A,B)** Frontal and lateral facial views show microcephaly, a broad forehead with bitemporal narrowing, sparse eyebrows, upslanting palpebral fissures, bilateral epicanthal folds, telecanthus, malar flattening, anteverted nares, a thin upper lip vermilion, downturned corners of the mouth, and low-set ears. **(C,D)** Examination of the extremities revealed persistent fetal fingertip pads, a single transverse palmar crease, abnormal plantar creases, and a sandal gap. **(E)** Sagittal brain magnetic resonance imaging showing corpus callosum thinning (arrow). **(F–I)** Follow-up photographs obtained at 3 years of age showing persistence of several craniofacial and extremity abnormalities. **(F,G)** Although the Down syndrome-like facial gestalt remains recognizable, it is less pronounced than in infancy. **(H,I)** Persistent palmar and plantar abnormalities are observed.

Additional findings included moderate conductive hearing loss in the right ear detected by brainstem auditory evoked potentials, myopia of −3.5 diopters, and seizures beginning at 9 months of age. Brain MRI revealed supratentorial ventriculomegaly, corpus callosum thinning (Figure 1E), a retrocerebellar arachnoid cyst, subcortical atrophy, diffuse periventricular gliosis, and reduced caliber of the right internal carotid artery. Echocardiography revealed an ostium secundum atrial septal defect.

Given the presence of several dysmorphic features, a clinical diagnosis of DS was initially suspected. Nevertheless, the phenotype was not fully consistent with classic DS, as several characteristic craniofacial and extremity features, including marked brachycephaly, pronounced epicanthal folds, and typical hand and foot anomalies were absent or mildly expressed in this case. Follow-up clinical photographs obtained at 3 years of age demonstrated persistence of several dysmorphic features, although the DS-like facial gestalt appeared less evident than during infancy (Figures 1F–I). At his last medical appointment, the milestones he had reached in his neurodevelopment included the following: beginning to use disyllabic words, using babbling and gestures to communicate, being able to walk unaided, having incomplete sphincter control, and his mother reporting hyperactive behaviour. The patient has received support from his mother and great-grandparents. He has good social relations and is receiving language therapy. He will continue receiving long-term care from different services.

## 3 Materials and methods

### 3.1 Ethical approval

The patient’s parents provided written informed consent for the genetic analyses and the publication of clinical, cytogenetic and molecular findings. All procedures were conducted in accordance with the principles of the Declaration of Helsinki and were approved by the Institutional Research and Ethics Committee. The study protocol has the approval reference 2017/040 INP and the Ethics Committee registration is CONBIOETICA-09-CEI-025-20161215.

### 3.2 Cytogenomic characterization

Cytogenetic analyses of the patient and his parents were performed on GTG-banded metaphases obtained from phytohemagglutinin (PHA)-stimulated peripheral blood lymphocytes according to standard procedures. Chromosome preparations were analyzed at a resolution of 450–550 bands, and 100 metaphases were evaluated and interpreted according to the International System for Human Cytogenomic Nomenclature (ISCN 2024) (Hastings et al., 2024).

Chromosomal microarray analysis was performed using the Affymetrix CytoScan® 750K Array platform (Thermo Fisher Scientific, Santa Clara, CA, United States) on genomic DNA extracted from peripheral blood. Array processing was performed according to the manufacturer’s protocol. Data were analyzed using Chromosome Analysis Suite (ChAS) software version 4.2. Copy number alterations were identified using log_2_ ratio and B-allele frequency plots, and mosaic proportions were estimated based on BAF distributions. Genomic coordinates were reported according to the GRCh37/hg19 genome assembly.

### 3.3 Ring chromosome structural configuration analysis

The following FISH probes were used individually or in combination to resolve the structural configuration of the ring chromosomes (Li et al., 2022): D13Z1/D21Z1 alpha-satellite probe (13/21 centromere; red signal) (Chen et al., 2012); LSI *RUNX1* probe (21q22.12; green signal); (Guzmán-Santiago et al., 2025); LSI DSCR (DSCR: D21S259/D21S341/D21S342, 21q22.13–q22.2; red signal); and (Zhang et al., 2012) 21q subtelomeric probe (VIJyRM 2029; yellow signal). The D13Z1/D21Z1 probe was manufactured by Cytocell (Tarrytown, NY, United States), whereas all remaining probes were produced by Abbott Molecular/Vysis (Chicago, IL, United States). FISH analyses were performed on metaphase chromosomes obtained from PHA-stimulated peripheral blood lymphocytes.

### 3.4 Tissue-specific dynamic mosaicism analysis

The composition and clonal distribution of ring chromosome-derived cell populations were evaluated by interphase FISH using the D13Z1/D21Z1 alpha-satellite probe (red) and the *RUNX1* probe (green). Analyses were performed on three tissues representing different embryonic germ layers: PHA-stimulated peripheral blood lymphocytes (PPBLs), oral mucosa epithelial cells (OMECs), and urinary sediment epithelial cells (USECs). Samples were collected using non-invasive procedures and processed according to standard laboratory protocols. A minimum of 100 nuclei per tissue were analyzed to determine the frequency and distribution of the different mosaic cell populations.

### 3.5 Database mining and literature review

We searched for reports describing a DS-like phenotype associated with a non-supernumerary r (21) carrying a 21q terminal deletion together with duplication of the DSCR (21q22.13–q22.3). A PubMed search was conducted through June 2026 using the following terms and combinations: “ring chromosome 21”, “ring AND chromosome 21”, “ring chromosome 21 AND deletion”, “ring chromosome 21 AND monosomy”, “ring chromosome 21 AND deletion AND duplication”, “ring chromosome 21 AND monosomy AND trisomy”, and “ring chromosome 21 AND constitutional”. The search was restricted to articles published between January 2000 and June 2026. Studies were excluded if they involved chromosomal imbalances outside 21q chromosome or if the cases lacked molecular cytogenetic characterization beyond conventional G-banded karyotyping.

### 3.6 Candidate dosage-sensitive selection

To investigate potential genotype–phenotype correlations, we selected both predicted and curated dosage-sensitive genes located within the recurrently deleted and duplicated regions of chromosome 21 identified in our patient and in previously reported cases. Genes within these regions were selected based on their predicted dosage sensitivity scores from DECIPHER (v11.38). This included genes with high predicted triplosensitivity (pTriplo ≥0.94) or haploinsufficiency (pHaplo ≥0.86), as well as ClinGen dosage sensitivity curations. Candidate genes were prioritized according to published evidence linking dosage imbalance to Human Phenotype Ontology (HPO) terms corresponding to the observed clinical manifestations in our patient and in comparable published cases.

Only protein-coding genes that met the predefined dosage sensitivity criteria were considered for downstream genotype–phenotype correlation analyses. For the purposes of this study, the classical DSCR was defined as the interval extending from the polymorphic marker D21S55 to the *MX1* locus, according to the original genotype–phenotype mapping studies by Delabar et al. (1993) and Sinet et al. (1994) (Delabar et al., 1993; Sinet et al., 1994). The highly restricted DSCR (HR-DSCR) was defined as the 34-kb interval spanning chr21:37,929,229–37,963,130 (GRCh37/hg19), according to Pelleri et al. (2016).

## 4 Results

### 4.1 Cytogenomic characterization

Conventional cytogenetic analysis revealed the following karyotype: mos 45,XY,-21 [3]/46,XY,der (21)dup r (21) (p11.1→q22.3→q22.3→q21.2)[86]/46,XY,idic r (21) (p11.1q22.3→q22.3q21.2p11.1q22.3→q22.3q21.2)[4]/46,XY,r (21) (p11.1q22.3)[7] in PPBLs from the proband (Figure 2A; Supplementary Table S1). The parents had normal karyotypes. SNP-array analysis identified an 18.77 Mb copy number gain at 21q21.2-q22.3 (chr21:26, 226, 101–44,996,871) and a 3.09 Mb terminal copy number loss at 21q22.3 (chr21:44,996,888-48,093,361) (Figure 2B). No additional clinically relevant copy number variants were detected. Although conventional cytogenetic analysis demonstrated mosaicism, the SNP-array classified chromosome 21 imbalances as non-mosaic copy number alterations (CN = 3 and CN = 1), likely reflecting the predominance of the der (21)dup r (21) cell line in peripheral blood. The SNP array results corresponded only to the clone with the duplicated ring, while the other clones were detected by G-banding and FISH. It is important to note that the cell types analyzed differ because these analyses were performed on PPBLs. In contrast, the SNP array was performed on non-stimulated blood, with genomic DNA primarily derived from myeloid lineage cells (Figure 2B; Supplementary Table S1).

**FIGURE 2:**
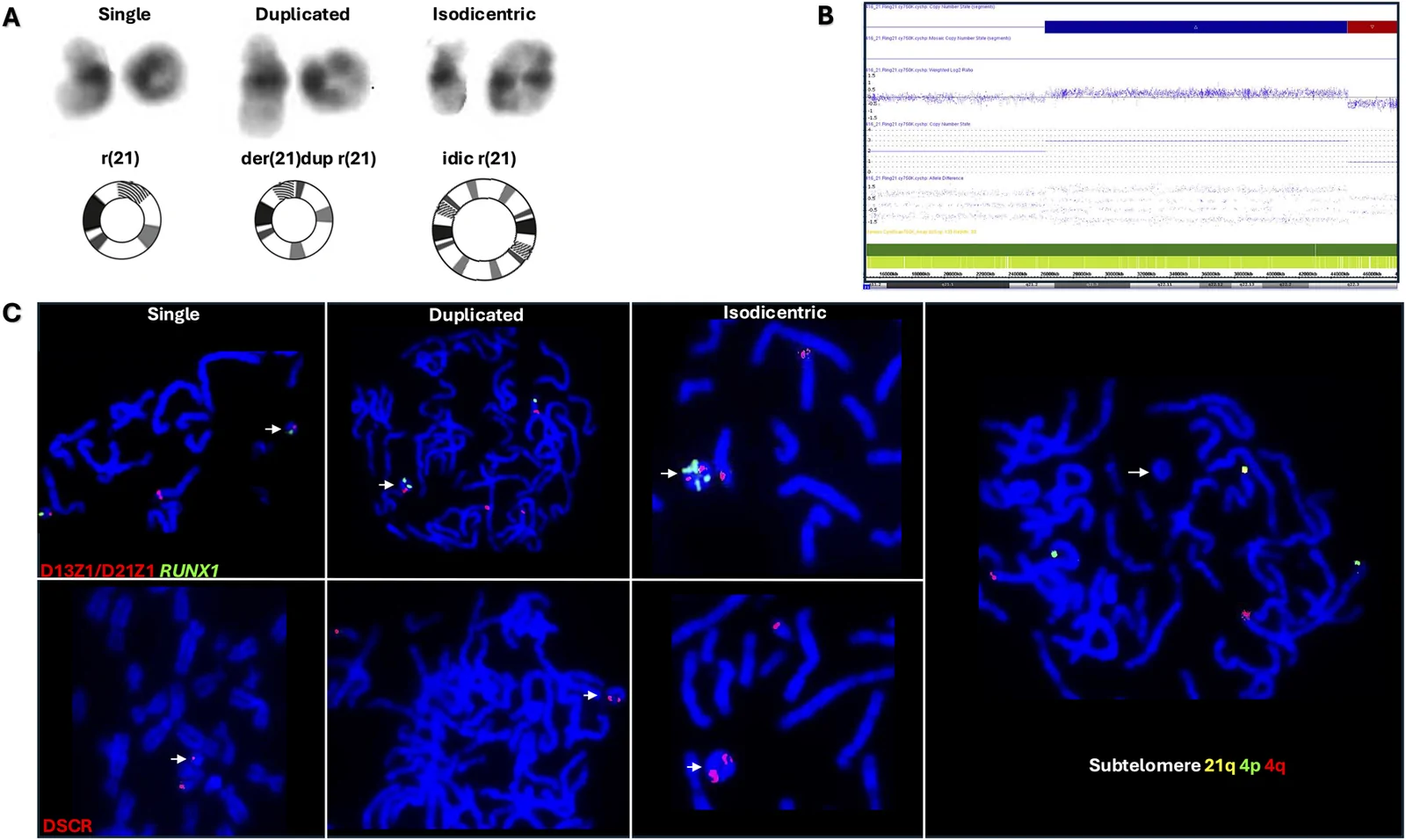
Cytogenomic characterization and structural reconstruction of the constitutional non-supernumerary ring chromosome 21. **(A)** Partial GTG-banded karyotypes and schematic representations of the three ring chromosome configurations identified in the proband: single ring r (21), duplicated monocentric ring der (21)dup r (21), and duplicated isodicentric ring idic r (21). **(B)** SNP-array profile of chromosome 21 showing an 18.77-Mb copy number gain involving 21q21.2-q22.3 (blue) and a 3.09-Mb terminal copy number loss involving 21q22.3 (red). Log_2_ ratio and B-allele frequency (BAF) plots are consistent with the identified duplication/deletion pattern. **(C)** Metaphase fluorescence *in situ* hybridization (FISH) analyses using probes targeting the chromosome 13/21 alpha-satellite sequence (D13Z1/D21Z1; red), the *RUNX1* locus (green), the Down syndrome critical region (DSCR; red), and the chromosome 21 subtelomeric region (21q; yellow) with chromosome 4 subtelomeric probes (4p, green; 4q, red) used as internal controls. White arrows indicate the ring chromosome configurations.

### 4.2 Ring chromosome structures and mosaic distribution

The structural configurations of the ring chromosomes were determined using G-banding patterns, SNP array results, and FISH analyses performed with commercially available probes. Furthermore, FISH assays targeting the chromosome 13/21 alpha-satellite sequence and the *RUNX1* locus enabled the characterization of the tissue-specific distribution of the mosaic cell populations. *RUNX1* was selected for ring characterization due to its location within the duplicated region and outside the deleted segment. This allowed discrimination between different ring chromosome-derived clones (Figure 2C; Supplementary Table S1). The structural compositions of the different rings detected are described as follows:

#### 4.2.1 Single ring

This clone was detected with low-level mosaicism (7%–9%) in all evaluated tissues (Supplementary Figures S1A,B). This structure results in partial monosomy 21, caused by the loss of the terminal 21q22.3 subtelomeric region.

#### 4.2.2 Duplicated monocentric ring

This clone is widely distributed among different tissues and represents the predominant cell line in PPBLs and OMECs (81%–86%) (Supplementary Figures S1A,B). The structure of this clone resulted in the coexistence of partial trisomy and partial monosomy of chromosome 21. This is caused by an inverted duplication of the 21q21.2-q22.3 segment, which includes *RUNX1* and the DSCR, as well as terminal deletion of 21q22.3.

#### 4.2.3 Isodicentric ring

This clone was detected with low-level mosaicism (2%–7%) in all evaluated tissues (Supplementary Figures S1A,B). The isodicentric ring resulted in partial pentasomy of 21q21.2-q22.3, as well as partial monosomy of 21q22.3, owing to the duplication of an already duplicated monocentric ring chromosome.

#### 4.2.4 Ring loss

This clone was observed with low frequency in PPBLs and OMECs (3%). In contrast, USECs exhibited this clone with a substantially higher frequency (50%) (Supplementary Figures S1A,B). The loss of the ring chromosome resulted in complete monosomy 21.

### 4.3 Literature review and genotype-phenotype correlation

The literature review identified five previously reported cases of constitutional non-supernumerary r (21) associated with concurrent 21q deletions and duplications involving the DSCR (Zhang et al., 2012; Chen et al., 2022; Murry and DuPont, 2025; Crombez et al., 2005). The case reported by Chen et al. (2022) corresponded to a 17-week-old fetus and was therefore excluded from the phenotypic comparison owing to the lack of a postnatal clinical evaluation (Chen et al., 2022). The remaining four cases involved pediatric patients younger than 18 months of age who carried constitutional r (21) and presented with multiple chromosome 21 genomic imbalances (Pelleri et al., 2016; Murry and DuPont, 2025; Crombez et al., 2005).

These patients share clinical features, including growth delay, global developmental delay, hypotonia, cranial dysmorphism, and ophthalmological, cardiac, and gastrointestinal abnormalities. In contrast, characteristic craniofacial features and limb anomalies typically observed in classic Down syndrome (DS) were absent or mildly expressed in most patients. Notably, our patient and the previously reported cases also presented with clinical manifestations that are uncommon in classic DS. These features include structural brain abnormalities (e.g., mild cerebral asymmetry, holoprosencephaly, and corpus callosum flattening), seizures, and genital anomalies (Table 1) (Zhang et al., 2012; Murry and DuPont, 2025; Crombez et al., 2005).

**TABLE 1:** Summary of genetic and clinical features in patients with Down Syndrome-like phenotype and non-supernumerary constitutional r (21) with complex genomic imbalances.

References	Crombez et al. (2005)	Zhang et al. (2012) Case #1	Zhang et al. (2012) Case #2	Murry et al. (2025)	Present case	Canonical DS phenotype
Predominant genomic imbalances associated to r (21)	q22.13q22.2 duplication Subtelomeric deletion	q22.12q22.13 duplication q22.2q22.3 deletion	q22.2q22.3 duplication q22.3 and q22.12q22.2 deletion	q22.11q22.13 and q22.3 Duplications q22.3 Deletion	q21.2q22.3 duplication q22.3 deletion	​
Duplication of DSCR						
Classic	Partial	Partial	Partial	Partial	✓	​
Highly restricted	✓	✗	✗	✗	✓	​
Age/Gender	Newborn/F	1 year/F	1.25 years/M	Newborn/M	8-months-old/M	​
Growth delay						
Short stature	✓	✗	✓	n.e	✓	✓
Low weight	✗	✗	✗	n.e	✗	✓
Neurological features						
GDD	✓	✓	✓	✓	✓	✓
Hypotonia	✓	✓	✗	n.e	✓	✓
Seizures	✗	✗	✓	n.e	✓	✗
Brain malformations	✓	✗	✗	✓	✓	✗
Cranial dysmorphism	✗	✓	✓	n.e	✓	✓
Facial dysmorphism						
Midface hypoplasia	Mild	✓	✗	n.e	Mild	✓
Upslanting palpebral fissures	Mild	✗	✗	n.e	✓	✓
Epicanthal folds	✗	✓	✗	n.e	✓	✓
Flat nasal bridge	Mild	✓	✗	n.e	✓	✓
Anteverted nares	Mild	✗	✗	n.e	✓	✓
Malar hypoplasia or micrognathia	Mild	✗	✓	n.e	Mild	✓
Mispositioned and/or dysmorphic ears	✗	✗	✓	n.e	✓	✓
Short neck	✗	✗	✗	n.e	✓	✓
Hearing loss	✗	✗	Mild	n.e	✓	✓
Ophthalmologic features	✗	✓	✓	n.e	✓	✓
Limb anomalies						
Brachydactyly	Mild	✓	✗	n.e	Mild	✓
Clinodactyly of 5th finger	✗	✗	✗	n.e	Mild	✓
Wide gap toes	Mild	✗	✗	n.e	✗	✓
Transverse palmar crease	✗	✗	✗	n.e	✓	✓
Joint hypermobility	✗	✓	✗	n.e	✓	✓
Cardiac anomalies	✓	✗	✓	n.e	✓	✓
Gastrointestinal anomalies	✓	✗	✗	n.e	✓	✓
Genital anomalies	✗	✗	✓	n.e	✗	✗
Others	​	​	Dental malocclusion Sleep apnea. Von Willebrand´s disease	Pulmonary hypertension	Spastic quadriparesis	​

F: Female. M: Male. DS: Down syndrome. DSCR: Down syndrome critical region. GDD: Global developmental delay. n.e: Non specified.

Comparative genomic analysis showed that all patients carried a complete or partial duplication of the classical DSCR. The duplicated interval consistently included the predicted triplosensitive genes *KCNJ6, DYRK1A,* and *ERG* (Table 1; Figure 3). In contrast, the copy number status of the HR-DSCR varied among cases. HR-DSCR duplication was identified in our patient, as well as in the case reported by Crombez et al. (2005) and in case 1 described by Zhang et al. (2012, case 1) (Zhang et al., 2012; Crombez et al., 2005). However, case 2, also described by Zhang et al. (2012), carried a deletion encompassing the HR-DSCR (Zhang et al., 2012). The patient reported by Murry et al. (2025) retained a normal copy number of HR-DSCR (Figure 3) (Murry and DuPont, 2025).

**FIGURE 3:**
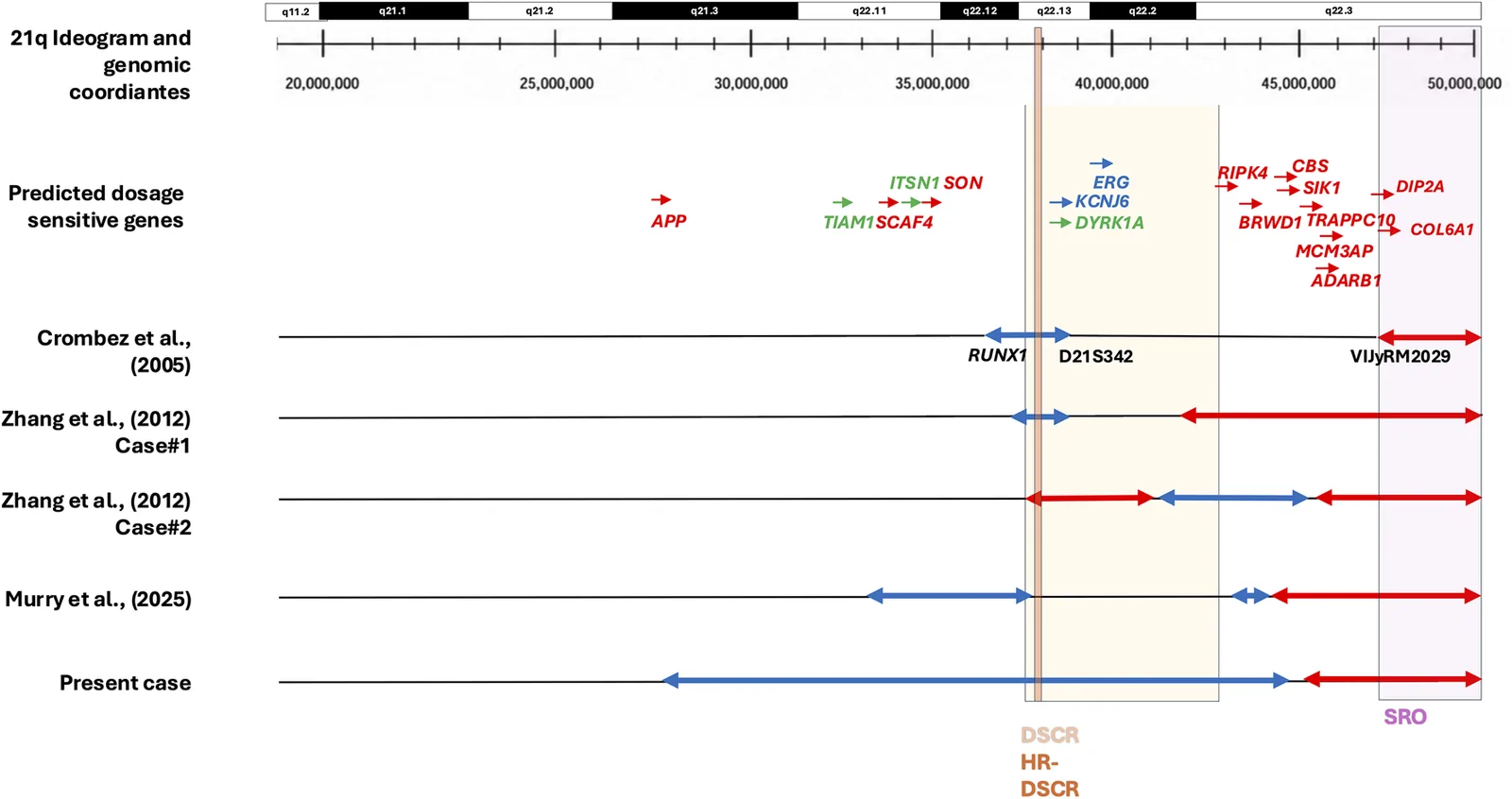
Comparative genomic imbalances and dosage-sensitive genes in the present patient and previously reported cases of constitutional non-supernumerary ring chromosome 21. A schematic representation of chromosome 21q, showing the cytobands and genomic coordinates (GRCh37/hg19) is presented at the top. Predicted dosage-sensitive genes are indicated by arrows positioned according to their genomic location and colored according to their predicted dosage sensitivity: haploinsufficient (red), triplosensitive (blue), and both haploinsufficient and triplosensitive (green). The duplicated (blue) and deleted (red) genomic intervals identified in the present case and the four comparable cases from the literature are shown according to their GRCh37/hg19 genomic coordinates. Because genomic coordinates were not available for the patient reported by Crombez et al. (2005), the duplicated and deleted intervals were inferred from the proximal and distal positions of the FISH probes used for molecular cytogenetic characterization. The classical Down Syndrome Critical Region (DSCR), the highly restricted Down Syndrome Critical Region (HR-DSCR), and the smallest region of overlap (SRO) of the terminal 21q22.3 deletions are highlighted in yellow, orange, and purple, respectively.

A comparison of the recurrently duplicated intervals identified additional predicted triplosensitive genes located outside the DSCR, including *TIAM1,* and *ITSN1*. Similarly, analysis of recurrent 21q22.3 terminal deletions identified *DIP2A* and *COL6A1* as the minimal set of predicted haploinsufficient genes shared among the reported cases (Figure 3; Supplementary Table S2).

## 5 Discussion

We report a new patient with a constitutional non-supernumerary r (21) carrying a large interstitial duplication harboring the DSCR and a terminal 21q22.3 deletion, presenting with a DS-like phenotype. Comprehensive cytogenetic and cytogenomic analyses not only characterized the genomic architecture of the ring chromosomes but also revealed four instability-associated cell clones across multiple tissues. Based on these findings, we propose the following sequence of events underlying the observed clonal diversity (Li et al., 2022): formation of the initial single r (21) involving terminal breaks in both chromosome arms and end-to-end fusion (Chen et al., 2012); intrachromosomal unequal recombination within the single ring, generating a duplicated monocentric r (21) with partial trisomy of 21q21.2-q22.3 region (Guzmán-Santiago et al., 2025); subsequent duplication of the duplicated monocentric ring, resulting in a duplicated idic r (21); and (Zhang et al., 2012) loss of the ring chromosome during mitosis as a consequence of its intrinsic instability.

In our patient, the presence of instability-derived clones across different tissues may have contributed to phenotypic variability through alterations in the dosage of genes on chromosome 21. The predominance of the complete monosomy 21 cell line in USECs suggests a marked tissue-specific ring chromosome instability within epithelial lineages. Urinary sediment contains exfoliated epithelial cells derived from the renal tubules, renal pelvis, ureters, bladder, and urethra (Dörrenhaus et al., 2000). Therefore, the high frequency of monosomic cells observed in USECs may reflect broader epithelial mosaicism rather than an alteration confined to the urinary tract alone.

Monosomic clones were also detected at low frequencies in PPBLs and OMECs. Previous studies have shown that mosaicism can be reliably identified using SNP arrays in samples with numerical abnormalities exceeding 20%. However, abnormalities present in approximately 10% of the cell population may still produce detectable deviations. Since additional tissues were not examined in this study, complete monosomy 21 or other cell lines derived from instability may be present in other tissues. However, it is important to note that several technical aspects of interphase FISH analysis of USECs may contribute to false-positive results. These aspects include the volume of the collected sample, the presence of different cell types, and the signal overlap within the nuclei. Therefore, findings suggestive of chromosomal abnormalities should be interpreted with caution and confirmed using an appropriate complementary method (Steventon-Jones et al., 2022).

Constitutional complete monosomy 21 is generally considered incompatible with postnatal survival, and all reported survivors exhibit mosaicism involving additional cell lines. Their phenotype typically includes severe developmental delay, growth restriction, microcephaly, craniofacial dysmorphism, congenital heart defects, hearing impairment, seizures, and structural brain abnormalities (Toral-Lopez et al., 2012). Previous studies have suggested that clinical severity depends primarily on the tissue distribution of monosomic cells rather than their proportion in peripheral blood (Toral-Lopez et al., 2012). Interestingly, several of these manifestations, including congenital heart disease, developmental delay, hearing impairment, and structural brain abnormalities, were also observed in our patient. Although these features are most likely explained by the combined effects of the 18.77-Mb 21q21.2-q22.3 duplication, terminal 21q22.3 deletion, and ring chromosome instability, the predominance of complete monosomy 21 in USECs raises the possibility that tissue-specific monosomic clones further modulate organ-specific phenotypic variability. This interpretation is consistent with previous reports of constitutional ring chromosome 21, in which the phenotype severity has been linked to the tissue distribution of instability-derived cell lines (Burgess et al., 2014).

The frequency of the duplicated monocentric r (21) was lower in epithelial cells than in PPBLs. This finding is consistent with the distinct proliferative behavior associated with origin of the analyzed tissue. Epithelial tissues undergo continuous physiological renewal, whereas peripheral blood T lymphocytes predominantly remain in a quiescent (G0) state until antigenic stimulation, resulting in substantially fewer cell divisions under physiological conditions (Squier and Kremer, 2001; Chapman et al., 2020). Consequently, the greater cumulative mitotic activity of epithelial tissues may partially explain the increased diversity of instability-derived clones, including the duplicated isodicentric r (21) and complete monosomy 21 (Supplementary Figure S1). These findings highlight the importance of multi-tissue cytogenomic analysis for revealing tissue-specific mosaicism and improving genotype–phenotype correlations in constitutional ring chromosome disorders.

Despite tissue-specific differences, the duplicated monocentric r (21) remained well-represented in all evaluated tissues. This finding indicates that the main genomic imbalance underlying the patient’s phenotype is a combination of partial trisomy of 21q21.2-q22.3 and terminal monosomy of 21q22.3.

The comparative analysis of this patient and previously reported cases suggests that copy number changes involving either the classical DSCR or the HR-DSCR alone are insufficient to explain the phenotypic variability observed among patients with constitutional non-supernumerary r (21). While all reported patients had a complete or partial duplication of the classical DSCR, the copy number status of the HR-DSCR varied considerably (Zhang et al., 2012; Murry and DuPont, 2025; Crombez et al., 2005). This variation included duplication, deletion, and normal copy numbers, despite broadly similar clinical manifestations (Table 1; Figure 3). The lack of concordance between HR-DSCR copy number and phenotype suggests that additional genomic factors contribute to the observed clinical variability.

Furthermore, recurrent genomic imbalances outside the DSCR consistently involve genes with varying levels of evidence for dosage sensitivity. These genes include *TIAM1, COL6A1, DIP2A, APP,* and *ITSN1* (Figure 3). According to the ClinGen dosage sensitivity curations, *DIP2A* and *ITSN1* have little/minimal evidence of haploinsufficiency (HI = level 1) and no evidence of triplosensitivity (TS = level 0). In contrast, *APP* has no evidence of haploinsufficiency (HI = level 0) and little/minimal evidence for triplosensitivity (TS = level 1). Currently, no ClinGen dosage sensitivity curation is available for *COL6A1* or *TIAM1* (Supplementary Table S2). Despite these differences in the strength of dosage sensitivity evidence, these genes have biological functions related to neuronal development and function, and their recurrent involvement across comparable patients suggests that alterations in their dosage may contribute to the neurological manifestations associated with constitutional non-supernumerary r (21). Collectively, these findings support the hypothesis that the phenotype associated with constitutional non-supernumerary r (21) cannot be explained solely by dosage alterations involving the classical or narrowly defined DSCR. Rather, it may reflect the combined effects of dynamic ring chromosome mosaicism and cumulative dosage imbalances involving multiple genes distributed across chromosome 21.

This retrospective, observational, descriptive, single-patient case study details the clinical presentation, diagnostic process, cytogenomic characterization, and implications for genetic counseling. After a thorough characterization of r (21) and cytogenetic evaluation of both parents, it was determined that the chromosomal abnormality was *de novo*. The patient’s mother received genetic counseling, which included an explanation of the nature of the chromosomal abnormality, its potential clinical implications, and the risk of recurrence. Since the parental cytogenetic studies were normal, the risk of recurrence was considered low. However, familial transmission of r (21) has been documented, including from a mosaic carrier parent (Bertini et al., 2008; Hertz, 1987). Therefore, a small residual recurrence risk cannot be excluded, especially because low-level or germline-limited parental mosaicism may not be detected by conventional cytogenetic analyses of peripheral blood. The mother demonstrated a clear understanding of the information provided, which was reinforced during subsequent follow-up visits to the Department of Human Genetics.

In conclusion, we report a new case of constitutional non-supernumerary r (21) associated with multiple genomic imbalances and a DS-like phenotype. The comprehensive cytogenetic and cytogenomic characterization of multiple tissues revealed tissue-specific clonal dynamics resulting from ring chromosome instability, emphasizing the importance of multi-tissue analysis for accurately assessing constitutional mosaicism. Integration of our findings with previously reported cases further suggests that dynamic ring chromosome mosaicism and cumulative dosage imbalances involving multiple chromosome 21 genes, both within and outside the classical DSCR, jointly shape the resulting phenotype. Further characterization of additional patients is essential to refine the clinical spectrum of this rare disorder and improve genotype–phenotype correlations.

## Data Availability

The original contributions presented in the study are included in the article/Supplementary Material, further inquiries can be directed to the corresponding authors.

## Supplementary material

SUPPLEMENTARY FIGURE S1 Tissue-specific distribution of ring chromosome-derived mosaic cell populations identified by interphase fluorescence *in situ* hybridization (FISH). **(A)** Representative interphase FISH nuclei illustrating the four-ring chromosome-derived cell populations identified in phytohemagglutinin-stimulated peripheral blood lymphocytes (PPBLs), oral mucosa epithelial cells (OMECs), and urinary sediment epithelial cells (USECs). Hybridization was performed using a chromosome 13/21 alpha-satellite probe (D13Z1/D21Z1; red) together with a *RUNX1* probe (green). Signal patterns allowed identification of the following clones: single ring chromosome (4 red, 2 green signals; partial monosomy 21q), duplicated monocentric ring chromosome (4 red, 3 green signals; partial trisomy/monosomy 21q), duplicated isodicentric ring chromosome (5 red, 5 green signals; partial pentasomy/monosomy 21q), and ring chromosome loss (3 red, 1 green signal; complete monosomy 21). Schematic diagrams illustrating the structural configuration of each clone are shown above the representative nuclei. **(B)** Distribution of the four-ring chromosome-derived cell populations across the three analyzed tissues.
