# Supplementary material for: Dynamic tissue-specific mosaicism in constitutional non-supernumerary ring chromosome 21 with a Down syndrome-like phenotype: a case report and genotype-phenotype analysis
Source: Front Genet. 2026 Oct 26;17:1958717. doi: 10.3389/fgene.2026.1958717 (PMC13744756; doi:10.3389/fgene.2026.1958717)

Supplementary Table 1. Summary of cytogenomic analysis performed in the proband

Mosaic ring clones	Metaphases from PBLs	Interphases from different tissues				21q genomic imbalances
	ISCN 2024 formula	PPBLs	OMECs	USECs	ISCN 2024 formula	
Single	G-bands<br>46,XY,r(21)(p11.1q22.3) [7/100]<br><br>FISH<br>ish r(21)(D13Z1/D21Z1+,*RUNX1*+,DSCR+,VIJyRM2029-)†	7%	9%	8%	nuc ish (D13Z1/D21Z1 x4, *RUNX1* x2) *	Partial monosomy
Duplicated	G-bands<br>46,XY,der(21)dup r(21)(::p11.1→q22.3→q22.3→q21.2::) [86/100]<br><br>SNParray *<br>arr[GRCh37]21q21.2q22.3(26,226,101-44,996,871)x3,21q22.3(44,996,888-48,093,361)x1<br><br>FISH<br>ish der(21)dup r(21)(D13Z1/D21Z1+,*RUNX1*++,DSCR++,VIJyRM2029-)†	86%	81%	40%	nuc ish (D13Z1/D21Z1 x4, *RUNX1* x3) *	Partial monosomy and trisomy

Isodicentric	G-bands: 46,XY,idic r(21)(p11.1q22.3→q22.3q21.2::p11.1q22.3→q22.3q21.2) [4/100]<br><br>FISH<br>ish idic r(21) (D13Z1/D21Z1++,*RUNX1*++++,DSCR ++++,VIJyRM2029-)†	4%	7%	2%	nuc ish (D13Z1/D21Z1 x5, *RUNX1* x5) *	Partial monosomy and pentasomy
Loss	G-bands: 45,XY,-21 [3/100]<br><br>FISH<br>ish 21 (D13Z1/D21Z1+,*RUNX1*+,DSCR+,VIJy RM2029+)*	3%	3%	50%	nuc ish (D13Z1/D21Z1 x3, *RUNX1* x1) *	Monosomy

DSCR markers: D21S259/D21S341/D21S342. * This corresponds to whole-cell nuclear signal counts. † Correspond to signals that are evaluated specifically on the ring chromosome.

Supplementary Table 2. Dosage-sensitive genes located within the duplicated and deleted regions in patients with r(21) and DS-like phenotype.

Chromosome 21cytoband	Gene	DECIPHER predicted dosage effect	ClinGen dosage sensitive curations (level of evidence)	Main biological function	HPO associations
q21.1	*USP25*	Haploinsufficient (pHaplo = 0.97)	Non curated	Deubiquitinating enzyme that hydrolyzes ubiquitin moieties conjugated to substrates and thus, functions in various biological processes including inflammation and immune response	NR
q21.1	*NRIP1*	Haploinsufficient (pHaplo = 0.90)	Non curated	Positive regulator of the circadian clock gene expression stimulates transcription of BMAL1, CLOCK and CRY1 by acting as a coactivator for RORA and RORC	Congenital onset
q21.13	*TTC3*	Haploinsufficient (pHaplo = 0.87)	Non curated	E3 ubiquitin-protein ligase which catalyzes the formation of 'Lys-48'-polyubiquitin chains	NR
q21.2	*BTG3*	Haploinsufficient (pHaplo = 0.93)	Non curated	Metalloprotease which cleaves aggrecan, a cartilage proteoglycan, at the '1938-Glu-Leu-1939' site (within the chondroitin	NR

				sulfate attachment domain), and may be involved in its turnover (By similarity)	
q21.3	*APP*	Haploinsufficient (pHaplo = 0.88)	Haploinsufficiency (0)<br><br>Triplosensitive (1)	Functions as a cell surface receptor and performs physiological functions on the surface of neurons relevant to neurite growth, neuronal adhesion and axonogenesis	Neurodevelopmental abnormality, abnormality of vision, seizure, intellectual disability, global developmental delay, abnormal cerebral vascular morphology, abnormality of mental function
q21.3	*CCT8*	Haploinsufficient (pHaplo = 0.96)	Non curated	Component of the chaperonin-containing T-complex (TRiC), a molecular chaperone complex that assists the folding of actin, tubulin and other proteins upon ATP hydrolysis	NR
q22.1	*GART*	Haploinsufficient (pHaplo = 0.92)	Non curated	Trifunctional enzyme that catalyzes three distinct reactions as part of the 'de novo' inosine monophosphate biosynthetic pathway	NR
q22.1	*SLC5A3*	Haploinsufficient (pHaplo = 0.94)	Non curated	Electrogenic Na(+)-coupled sugar symporter that actively transports myo-inositol and its	NR

				stereoisomer scyllo-inositol across the plasma membrane, with a Na(+) to sugar coupling ratio of 2:1	
q22.1	*IFNGR2*	Haploinsufficient (pHaplo = 0.93)	Non curated	Ligand binding stimulates activation of the JAK/STAT signaling pathway	NR
q22.1	*HUNK*	Haploinsufficient (pHaplo = 0.96)	Non curated	Enables ATP binding	NR
q22.11	*TIAM1*	Triplosensitive (pTriplo = 0.96)<br><br>Haploinsufficient (pHaplo = 0.98)	Non curated	RAC1 guanine nucleotide exchange factor involved in neuronal migration, dendritic growth, and synaptic development	Growth delay, axial hypotonia, thin corpus callosum, cryptorchidism, global developmental delay
q22.11	*SCAF4*	Haploinsufficient (pHaplo = 0.99)	Non curated	Anti-terminator protein required to prevent early mRNA termination during transcription	Seizure, hypotonia, intellectual disability, global developmental delay, joint hypermobility, hypoplasia of the corpus callosum, congenital onset, epicanthus, microcephaly, high palate
q22.11	*PAXBP1*	Haploinsufficient (pHaplo = 0.99)	Non curated	Associates with a histone methyltransferase complex that specifically mediates dimethylation and trimethylation of 'Lys-4' of histone H3	NR
q22.11	*ITSN1*	Triplosensitive (pTriplo = 1)	Triplosensitive (0)	Adaptor protein involved in endocytosis, synaptic	Synaptic dysfunction, intellectual disability

		Haploinsufficient (pHaplo = 1)	Haploinsufficiency (1)	vesicle trafficking, and intracellular signaling	candidate gene, neurodevelopmental abnormalities
q22.11	*SON*	Haploinsufficient (pHaplo = 1)	Haploinsufficiency (0)<br><br>Triplosensitive (3)	RNA-binding protein that acts as a mRNA splicing cofactor by promoting efficient splicing of transcripts that possess weak splice sites	Myopia, intestinal atresia, abnormal brain morphology, depressed nasal bridge, atrial septal defect, patent ductus arteriosus, low set ears, hearing loss, growth delay, failure to thrive, high palate, epicanthus, ventriculomegaly, hypoplasia of the corpus callosum, midface retrusion, abnormality of the genitourinary system, floppy infant, neonatal hypotonia, joint hypermobility, abnormal cerebral white matter morphology, global developmental delay, intellectual disability, hypotonia, seizure, short stature, ventricular septal defect
q22.12	*MORC3*	Triplosensitive (pTriplo = 0.99)	Non curated	Nuclear matrix protein which forms MORC3-NBs (nuclear bodies) via an ATP-dependent mechanism and plays a role in innate immunity by restricting different viruses	NR

				through modulation of the IFN response	
q22.13	*KCNJ6*	Triplosensitive (pTriplo = 0.96)	Triplosensitive (0)<br><br>Haploinsufficiency (0)	G protein-gated inwardly rectifying potassium channel regulating neuronal excitability	Seizure, intellectual disability, global developmental delay, congenital onset, microcephaly, high palate, failure to thrive, spastic tetraparesis
q22.13	*DYRK1A*	Triplosensitive (pTriplo = 0.98)<br><br>Haploinsufficient (pHaplo = 1)	Triplosensitive (0)<br><br>Haploinsufficiency (3)	Serine/threonine kinase involved in neurodevelopment, neuronal differentiation, synaptic plasticity, and brain growth	Hypoplasia of the corpus callosum, abnormality of the genitourinary system, thin corpus callosum, cryptorchidism, brain imaging abnormality, global developmental delay, intellectual disability, seizure, generalized hypotonia, ventriculomegaly, gliosis, congenital onset, duodenal atresia, sleep disturbances, short stature, microcephaly, thin upper lip vermilion, failure to thrive, hearing impairment, low set ears, patent ductus arteriosus, upslanted palpebral fissure, myopia, ventricular septal defect
q22.2	*ERG*	Triplosensitive (pTriplo = 0.94)	Non curated	ETS-family transcription factor involved in	Hematopoietic abnormalities, leukemia susceptibility associated with trisomy 21

				hematopoiesis and vascular development	
q22.13	*SIM2*	Haploinsufficient (pHaplo = 0.98)	Non curated	Transcription factor that may be a master gene of CNS development in cooperation with Arnt	NR
q22.2	*BRWD1*	Haploinsufficient (pHaplo = 0.99)	Non curated	Plays a role in the regulation of cell morphology and cytoskeletal organization	Abnormality of the genitourinary system, morphological central nervous system abnormality, ventriculomegaly
q22.2	*DSCAM*	Haploinsufficient (pHaplo = 0.98)	Triplosensitive (0)<br><br>Haploinsufficiency (2)	Cell adhesion molecule that plays a role in neuronal self-avoidance	NR
q22.2	*BACE2*	Haploinsufficient (pHaplo = 0.95)		Responsible for the proteolytic processing of the amyloid precursor protein (APP)	NR
q22.2	*ETS2*	Haploinsufficient (pHaplo = 0.94)	Non curated	Binds specifically the DNA GGAA/T core motif (Ets-binding site or EBS) in gene promoters and stimulates transcription	NR
q22.3	*U2AF1*	Triplosensitive (pTriplo = 0.97)	Non curated	Plays a critical role in both constitutive and enhancer-dependent splicing by mediating protein-protein interactions and protein-RNA interactions required	NR

				for accurate 3'-splice site selection	
q22.3	*COL6A1*	Haploinsufficient (pHaplo = 0.94)	Non curated	Extracellular matrix collagen involved in muscle and connective tissue integrity	Failure to thrive, high palate, skin rash, generalized hypotonia, intellectual disability, joint hypermobility
q22.3	*DIP2A*	Haploinsufficient (pHaplo = 0.95)	Haploinsufficiency (1)<br><br>Triplosensitive (0)	Neurodevelopmental protein involved in axonal growth and neuronal connectivity	Developmental delay, intellectual disability, autism spectrum disorders
q22.3	*TRAPPC10*	Haploinsufficient (pHaplo = 0.98)	Haploinsufficiency (0)<br><br>Triplosensitive (0)	Specific subunit of the TRAPP (transport protein particle) II complex, a highly conserved vesicle tethering complex that functions in late Golgi trafficking as a membrane tether	Upslanted palpebral fissure, growth delay, thin upper lip vermilion, microcephaly, seizure, hypotonia, global developmental delay, ventriculomegaly, short stature
q22.3	*SIK1*	Haploinsufficient (pHaplo = 0.93)	Non curated	Serine/threonine-protein kinase involved in various processes such as cell cycle regulation, gluconeogenesis and lipogenesis regulation, muscle growth and differentiation and tumor suppression	Abnormality of the nervous system, EEG abnormality, hypoplasia of the corpus callosum, floppy infant, global developmental delay, intellectual disability, seizure, microcephaly, failure to thrive, depressed nasal bridge, anteverted nares
q22.3	*RIPK4*	Haploinsufficient (pHaplo = 0.92)	Associated with autosomal recessive phenotype (30)	Required for embryonic skin development and correct skin homeostasis in adults, via phosphorylation	Upslanted palpebral fissure, congenital onset, bilateral cryptorchidism, intellectual disability, motor delay,

			Triplosensitive (0)	of PKP1 and subsequent promotion of keratinocyte differentiation and cell adhesion	microcephaly, low set ears, microphtalmia, depressed nasal bridge, micrognatia
q22.3	*TMPRSS2*	Haploinsufficient (pHaplo = 0.93)	Non curated	Plasma membrane-anchored serine protease that cleaves at arginine residues 4	NR
q22.3	*C2CD2*	Haploinsufficient (pHaplo = 0.90)	Non curated	Target for miRNA	NR
q22.3	*ADARB1*	Haploinsufficient (pHaplo = 0.89)	Non curated	RNA-editing enzyme essential for neuronal maturation and neurotransmission	Epilepsy, upslanted palpebral fissure, high palate, thin upper lip vermilion, microcephaly, delayed ability to walk, short stature, hypoplasia of the corpus callosum, axial hypotonia, global developmental delay
q22.3	*MCM3AP*	Haploinsufficient (pHaplo = 0.88)	Non curated	DNA replication and nuclear transport regulator with roles in neuronal function	Intellectual disability, developmental delay, motor delay, congenital onset, short stature, delayed ability to walk, delayed speech and language development, obstructive sleep apnea
q22.3	*PCBP3*	Haploinsufficient (pHaplo = 0.96)	Non curated	RNA-binding protein involved in post-transcriptional gene regulation	Limited evidence; proposed neurodevelopmental role
q22.3	*PDE9A*	Haploinsufficient (pHaplo = 0.89)	Non curated	Specifically hydrolyzes the second messenger cGMP,	NR

				which is a key regulator of many important physiological processes	
q22.3	*AGPAT3*	Haploinsufficient (pHaplo = 0.88)	Non curated	Converts 1-acyl-sn-glycerol-3-phosphate (lysophosphatidic acid or LPA) into 1,2-diacyl-sn-glycerol-3-phosphate (phosphatidic acid or PA) by incorporating an acyl moiety at the sn-2 position of the glycerol backbone	NR
q22.3	*CBS*	Haploinsufficient (pHaplo = 0.88)	Associated with autosomal recessive phenotype (30)<br><br>Triplosensitive (0)	Hydro-lyase catalyzing the first step of the transsulfuration pathway, where the hydroxyl group of L-serine is displaced by L-homocysteine in a beta-replacement reaction to form L-cystathionine, the precursor of L-cysteine	Myopia, brain atrophy, neurodevelopmental delay, childhood onset, autistic behavior, seizure, intellectual disability, global developmental delay, abnormal cerebral white matter morphology, EEG abnormality, neonatal onset
q22.3	*ICOSLG*	Haploinsufficient (pHaplo = 0.87)	Non curated	Acts as a costimulatory signal for T-cell proliferation and cytokine secretion	NR
q22.3	*PRDM15*	Haploinsufficient (pHaplo = 0.86)	Non curated	Plays a role as a molecular node in a transcriptional network regulating embryonic development and cell fate decision	NR

q22.3	*RRP1B*	Haploinsufficient (pHaplo = 0.86)	Non curated	Positively regulates DNA damage-induced apoptosis by acting as a transcriptional coactivator of proapoptotic target genes of the transcriptional activator E2F1	NR

Supplementary Figure 1.

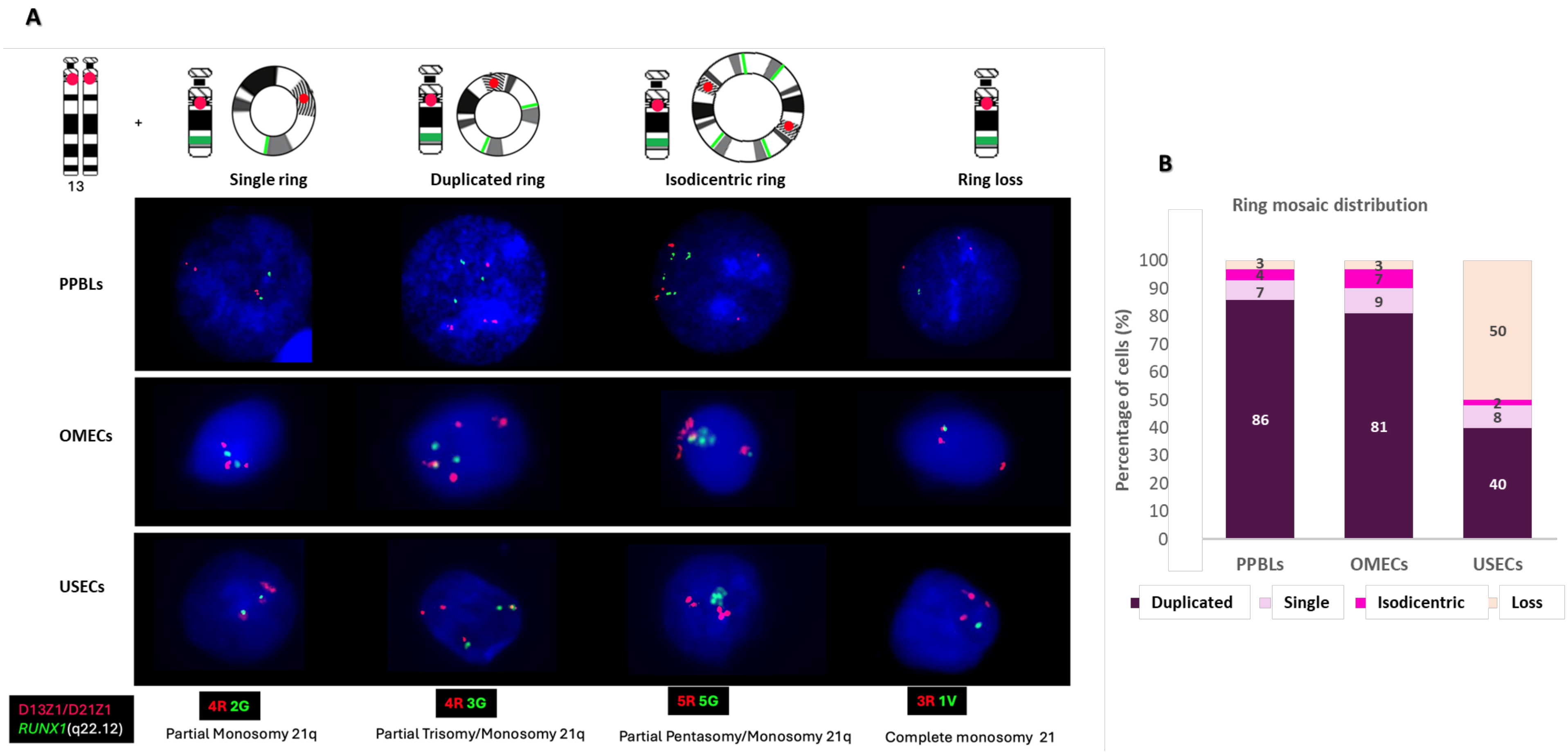

Supplement: Supplementary file 1 [file Supplementaryfile1.pdf]
